# Cholinergic Signaling Attenuates Pro-Inflammatory Interleukin-8 Response in Colonic Epithelial Cells

**DOI:** 10.3389/fimmu.2021.781147

**Published:** 2022-01-06

**Authors:** Isabelle Müller, Urs Kym, Virginie Galati, Sasha Tharakan, Ulrike Subotic, Thomas Krebs, Eleuthere Stathopoulos, Peter Schmittenbecher, Dietmar Cholewa, Philipp Romero, Bertram Reingruber, NIGStudy Group, Isabella Bielicki, Stefan Holland-Cunz, Simone Keck

**Affiliations:** ^1^ Department of Pediatric Surgery, University Children’s Hospital Basel (UKBB) and University of Basel, Basel, Switzerland; ^2^ Department of Pediatric Surgery, University Children’s Hospital Zürich, Zürich, Switzerland; ^3^ Department of Pediatric Surgery, Children’s Hospital of Eastern Switzerland, St. Gallen, Switzerland; ^4^ Department of Pediatric Surgery, University Hospital of Lausanne (CHUV), Lausanne, Switzerland; ^5^ Department of Pediatric Surgery, Municipal Hospital, Karlsruhe, Germany; ^6^ Department of Pediatric Surgery, Inselspital, Bern University Hospital, University of Bern, Bern, Switzerland; ^7^ Department of Pediatric Surgery, University Hospital of Heidelberg, Heidelberg, Germany; ^8^ Department of Pediatric Surgery, Florence Nightingale Hospital, Düsseldorf, Germany; ^9^ Department of Pathology, University Hospital of Lausanne (CHUV) and University of Lausanne, Lausanne, Switzerland

**Keywords:** Hirschsprung disease, Hirschsprung-associated enterocolitis, intestinal epithelial cell, Interleukin-8, acetylcholine receptors

## Abstract

Infants affected by Hirschsprung disease (HSCR), a neurodevelopmental congenital disorder, lack ganglia of the intrinsic enteric nervous system (aganglionosis) in a variable length of the colon, and are prone to developing severe Hirschsprung-associated enterocolitis (HAEC). HSCR patients typically show abnormal dense innervation of extrinsic cholinergic nerve fibers throughout the aganglionic rectosigmoid. Cholinergic signaling has been reported to reduce inflammatory response. Consequently, a sparse extrinsic cholinergic innervation in the mucosa of the rectosigmoid correlates with increased inflammatory immune cell frequencies and higher incidence of HAEC in HSCR patients. However, whether cholinergic signals influence the pro-inflammatory immune response of intestinal epithelial cells (IEC) is unknown. Here, we analyzed colonic IEC isolated from 43 HSCR patients with either a low or high mucosal cholinergic innervation density (fiber-low versus fiber-high) as well as from control tissue. Compared to fiber-high samples, IEC purified from fiber-low rectosigmoid expressed significantly higher levels of IL-8 but not TNF-α, IL-10, TGF-β1, Muc-2 or tight junction proteins. IEC from fiber-low rectosigmoid showed higher IL-8 protein concentrations in cell lysates as well as prominent IL-8 immunoreactivity compared to IEC from fiber-high tissue. Using the human colonic IEC cell line SW480 we demonstrated that cholinergic signals suppress lipopolysaccharide-induced IL-8 secretion *via* the alpha 7 nicotinic acetylcholine receptor (a7nAChR). In conclusion, we showed for the first time that the presence of a dense mucosal cholinergic innervation is associated with decreased secretion of IEC-derived pro-inflammatory IL-8 in the rectosigmoid of HSCR patients likely dependent on a7nAChR activation. Owing to the association between IL-8 and enterocolitis-prone, fiber-low HSCR patients, targeted therapies against IL-8 might be a promising immunotherapy candidate for HAEC treatment.

## Introduction

Hirschsprung disease (HSCR) is a multi-genetic congenital malformation of the colon characterized by the absence of enteric ganglia (aganglionosis) (1–4). Defects in genes involved in neuronal migration prevent neural crest cells from reaching the colon during fetal development. Consequently, aganglionosis affects a distal intestinal segment of variable length resulting in disturbed peristalsis, leading to chronic stool obstruction and formation of a megacolon. Most newborn HSCR-patients are diagnosed within 48h after birth, due to failing to pass the meconium. Therapeutic pull-through surgery is usually performed in the first month of life and involves resection of affected aganglionic tissue followed by the pull-through and distal anastomosis of healthy tissue (5). Hirschsprung-associated enterocolitis (HAEC) is the most severe complication and leading cause of morbidity in Hirschsprung disease (1, 6, 7). The underlying pathogenesis is not fully explored but involves changes in innate and adaptive immune cells (4, 8–10), microbial composition (4, 11–14) and epithelial barrier functions (2, 15–17).

In aganglionic rectosigmoid tissue from HSCR patients, acetylcholinesterase (AChE) activity is increased due to the presence of extrinsic AChE-positive nerve fibers innervating the muscularis, submucosa and the mucosa (4, 5, 18–20). Their histopathological evaluation in a rectal biopsy is supportively used to diagnose HSCR (5, 21). AChE is an enzyme responsible for the degradation of acetylcholine, the primary neurotransmitter of the parasympathetic nervous system as well as different types of enteric neurons. Acetylcholine (ACh) binds to two groups of receptors: the muscarinic and nicotinic ACh receptors (mAChR and nAChR). A variety of both are found in multiple cells including immune cells and intestinal epithelial cells (22, 23). Borovikova and colleagues described an anti-inflammatory effect of cholinergic signaling and introduced the term “cholinergic anti-inflammatory pathway” (CAIP) (24, 25). In a model of systemic inflammation, vagus nerve stimulation triggered the release of ACh which bound to alpha-7 nAChR (a7nAChR)-positive splenic macrophages and significantly attenuated the release of pro-inflammatory cytokines. This concept was extended to intestinal inflammation models using vagus nerve stimulation or pharmacological targeting of a7nAChR (26–29).

The gastrointestinal epithelial lining represents the frontline of host defence. Intestinal epithelial cells (IEC) are exposed to pathogenic and non-pathogenic antigens derived from harmful invaders or harmless commensals or food. Towards the luminal side IEC protect the host by the secretion of mucus and antimicrobial peptides. To maintain mucosal homeostasis, IEC sense intestinal signals *via* pattern-recognition receptors and coordinate the underlying immune system towards tolerance or activation (30). The recognition of commensal-derived antigens leads to an anti-inflammatory immune response establishing anti-commensal tolerance. This involves the secretion of anti-inflammatory cytokines [e.g., interleukin (IL)-10 and TGF-β1]. Loss of the epithelial barrier leads to the trespassing of pathogens, triggering an inflammatory immune response (e.g., IL-8 and TNFα). IL-8 (CXCL8) is a polymorphonuclear leukocyte chemokine that is rapidly secreted from IEC in response to bacterial stimuli and initiates influx of neutrophil into the mucosa, supporting T helper 17 (TH17) CD4 T cell differentiation (31, 32). IL-8 has been used in multiple studies as an indicator of intestinal inflammatory response (33–36). Consequently, dysregulated expression of IL-8 might contribute to the pathophysiology of HAEC, thus its targeting may have therapeutic benefit.

Previous work from our group uncovered a before-unknown correlation between the mucosal fiber innervation density and the onset of enterocolitis in HSCR patients (4). HSCR patients showing prominent colonic mucosal AChE^+^ nerve fibers (fiber-high) had decreased inflammatory cytokines and TH17 T cells as well as lower incidence of enterocolitis development compared to HSCR patients with only sparse mucosal innervation (fiber-low) (4, 37). AChE^+^ nerve fibers were closely associated with bipolar tissue-resident macrophages and their presence correlated with lower macrophage-dependent expression of the TH17-promoting cytokine IL-23. Macrophage-specific nicotinic (CHRNA7, CHRNA5, CHRNA1) and muscarinic (CHRM3) cholinergic receptors might be involved in neuronal immune cell modulation (4).

IEC have been reported to express cholinergic receptors and their essential role in maintaining barrier functions has been described (38). However, the impact of ACh signaling on epithelial cytokine production is not known.

Here we investigated the association of mucosal cholinergic innervation with cytokine expression of HSCR and Non-HSCR derived IEC and identified *in vitro* possibly involved cholinergic receptors and signaling pathways using the human SW480 IEC cell line.

## Materials and Methods

### Patients and Human Specimen

Pediatric patients with Hirschsprung disease as well as miscellaneous intestinal diseases were enrolled in a prospective Swiss- and German-wide multicenter study approved by the North-West Switzerland Ethics Commission (EKNZ 2015-049) and the Medical Faculty of Heidelberg (S-388/2015). The study is registered at www.clinicaltrials.gov under access number NCT03617640 and is performed according to the Declaration of Helsinki. Colonic tissue from consenting patients was collected freshly in the operating room. For histological analysis a longitudinal strip of resected colonic tissue was immediately snap-frozen. Tissue for epithelial cell isolation was kept on ice in transport medium (Hank’s Balanced Salt Solution (HBSS) supplemented with 10% Foetal Calf Serum (FCS, BioConcept AG, Allschwil, Switzerland), 10mM Hepes (ThermoFisher Scientific, Waltham, MA, USA), 1% Penicillin-Streptomycin, 0.5µg/ml amphotericin B, 80µg/ml gentamicin [all from Merck, Darmstadt, Germany)] and was processed in less than 24h. Demographic and clinical patient data were extracted from medical records. The Non-HSCR patient group included five patients with anal atresia and one misdiagnosed HSCR patient. Tissue was collected during ostomy and pull-through resection, respectively. The control tissue showed the presence of an ENS and the absence of inflammation as defined by the local pathologists. A detailed patients characteristic is available in Keck et al. (4).

### Isolation of Intestinal Epithelial Cells

Colonic tissue was separated into anatomical segments according to rectosigmoid and descending colon (distal to proximal) and muscle layers stripped from mucosa. Mucosa was minced and digested with collagenase D (1mg/ml) and DNase I (4µg/ml, both Roche Diagnostics, Basel, Switzerland) in complete medium (RPMI 1640 (BioConcept AG, Allschwil, Switzerland) supplemented with 10% FCS, 1% Penicillin-Streptomycin, 0.5µg/ml amphotericin B, 80µg/ml gentamicin, 10mM Hepes) at 37°C for 1h under vigorous shaking. Digestion suspension was filtered through a 100µm-cell strainer, and cells were washed in complete medium. Suspension cells were resuspended in 20% Percoll (Merck, Darmstadt, Germany) and underlaid with 40% Percoll (3ml of each). Epithelial cells were purified from the 20%/40% Percoll interface. Cells were washed and used freshly for Fluorescence-activated cell sorting (FACS) analysis or immediately resuspended in RLT Lysis buffer and cryo-stored until further analysis.

### Fluorescence-Activated Cell Sorting

Viability was determined using the LIVE/DEAD Fixable Near-IR Dead cell stain (ThermoFisher Scientific, Waltham, MA, USA). Epithelial purity was determined using human specific antibodies against CD45 (1:100; PerCP, HI30, Biolegend, San Diego, CA, USA) and EpCAM (1:500, Alexa 488, VU1D9, Cell Signaling Technology, Danvers, MA, USA). Cell surface markers were stained extracellular in 50µl FACS buffer (PBS supplemented with 2% FCS) for 15min on ice. Unspecific Fcγ receptor binding was blocked by human Fc block (CD16/CD32, BD Bioscience, Franklin Lakes, New Jersey, USA). Cells were analyzed using a FACS Canto II (BD Bioscience, Franklin Lakes, New Jersey, USA) and FlowJo software (TreeStar Inc.).

### RNA Isolation and Quantitative Real-Time Polymerase Chain Reaction

Total RNA from cells was isolated using the RNAeasy Plus mini kit (QiAgen, Hilden, Germany) and reverse transcribed using GoScript Reverse Transcription System (Promega, Madison, Wisconsin, USA). Quantitative Real-Time PCR (qRT-PCR) was done using the ViiA7 RT-PCR System (Thermo Fisher Scientific, Waltham, MA, USA) and FastStart Universal SYBR Green Master (Roche, Basel, Switzerland) according to manufacturer’s instruction. Relative gene expression was calculated using the 2^-ΔCT^ method, with β2-microglobulin as the housekeeping gene, and results were multiplied by 1000. Primer pairs were designed using the clone manager software (Sci-Ed Software). The following primer pairs were used (all from Microsynth, Balgach, Switzerland): β2-microglobulin: Forward: 5’-CAG CGT ACT CCA AAG ATT CA-3’ Reverse: 5’-GAA TGC TCC ACT TTT TCA AT-3’; IL-8: Forward: 5’-GTG CAG TTT TGC CAA GGA GT-3’ Reverse: 5’-CTC TGC ACC CAG TTT TCC TT-3’; TNFα: Forward: 5’-GCT GCA CTT TGG AGT GAT CG-3’ Reverse: 5’-GGG CTA CAG GCT TGT CAC TC-3’; TGFβ1: Forward: 5’-ATT GAG GGC TTT CGC CTT AG-3’ Reverse: 5’-AAT GGT GGC CAG GTC ACC TC-3’; IL10: Forward: 5’-GCC TTC AGC AGA GTG AAG AC-3’ Reverse: 5’-GGC TTG GCA ACC CAG GTA AC-3’; MUC2: Forward: 5’-GCC CTT GCG TCC ATA ACA AC-3’ Reverse: 5’-GTA GTA CTT CCC GTC AAA GG-3’; Occludin: Forward: 5’-ATG GAC TGC GTC ACG CAG AG-3’ Reverse: 5’-CCA CAA ACA TGG CCA GGA AG-3’; ZO-1: Forward: 5’-AGC ACA GCA ATG GAG GAA AC-3’ Reverse: 5’-GGT CCT CCT TTC AGC ACA TC-3’; CHRNA3: Forward: 5’- TGC TGT CTC TGC TGC GAC TG-3’ Reverse: 5’-ACA TGG ACA CCT CGA AAT GG-3’; CHRNA4: Forward: 5’-GGA GCG GCT CCT GAA GAA AC-3’ Reverse: 5’-CAC TCC TGC TTC ACC CAT AC-3’; CHRNA5: Forward: 5’-CAC ACT CAG TGC TCC ATT CC-3’ Reverse: 5’-CGA ACC CAT CTT TCG TAG TC-3’; CHRNA7: Forward: 5’-GAT GGC CAG ATT TGG AAA CC-3’ Reverse: 5’-CGA TGT AGC AGG AAC TCT TG-3’; CHRM1: Forward: 5’-GGC AGT GCT ACA TCC AGT TC-3’ Reverse: 5’-CGT GCT CGG TTC TCT GTC TC-3’; CHRM3: Forward: 5’- TCC GAG CAG ATG GAC CAA GAC-3’ Reverse: 5’- CTG TGA CCC GGA AGC TTG AG-3’.

### Immunohistochemistry

Longitudinal colonic strips were washed in ice-cold PBS and embedded as a Swiss Roll in Tissue-Tek O.C.T. Compound (Sakura, Osaka, Japan). Cholinergic nerve fibers were visualized in 5µm cryosections using a purified mouse IgG2b anti-human acetylcholinesterase antibody (HR2, Abcam) or a purified mouse IgG2a anti-human β3tubulin (2G10, Abcam, Cambridge, England) together with an anti-mouse HRP-AEC staining kit (R&D Systems, Minneapolis, Canada) according to the instruction manual. Slides were scanned with an Olympus BX63 Light microscope. The density of cholinergic fibers in epithelial regions of the rectosigmoid was evaluated by four double-blinded individuals and has been previously described in detail (4).

### Immunofluorescence

Between all steps, tissue was washed three times with PBS supplemented with 0.2% Tween-20 (Merck, Darmstadt, Germany). Cryosections (5µm) were fixed with 4% paraformaldehyde (PFA, ThermoFisher Scientific, Waltham, MA, USA) for 5min, followed by a blocking step with goat serum (40min). Primary human antibodies, purified mouse IgG1 anti-IL-8 (1:500; 3IL8-H10, ThermoFisher Scientific, Waltham, MA, USA), purified mouse IgG1 anti-alpha7nAchR (1:200; 836701, Biolegend, San Diego, CA, USA) and FITC-labeled mouse IgG2b anti-EpCAM (1:100; 9c4, BioLegend, San Diego, CA, USA) were incubated for 1h at room temperature. Purified primary antibodies were detected by secondary antibody goat anti-mouse IgG1 A555 (1:2000; ThermoFisher Scientific, Waltham, MA, USA) for 40min. Antibodies were diluted in Antibody-Diluent (Agilent, Santa Clara, CA, USA). Finally, nuclei were visualized by 4′,6-Diamidin-2-phenylindol (DAPI) staining (3min; 0.5µg/mL; ThermoFisher Scientific, Waltham, MA, USA), and slides were mounted using Prolong Diamond Antifade Mountant (ThermoFisher Scientific, Waltham, MA, USA). Secondary antibody controls were included as negative controls. Images were taken with a fluorescence microscope (BX43, Olympus, Shinjuku, Japan) and analyzed using CellSense software (Olympus).

For the quantification of IL-8, images were analyzed using Cellprofiler-3.1.9 software. To quantify IL-8 expression in epithelial cells in the two patient groups (fiber-low versus fiber-high, n=3), 4 to 8 epithelial regions (in average 1500 IEC) per patient from the rectosigmoid were examined. Primary objects were defined by nuclear DAPI staining. Secondary objects were defined by extracellular EpCam expression using the watershed propagation module after applying a global threshold to identify epithelial cells. IL-8-positive cells were identified as a second primary object using Three Classes Otsu thresholding method, and subsequently related to EpCam^+^ cells. Frequencies of IL-8^+^ EpCam^+^ cells were shown as the percentage of total EpCam^+^ cells. Patients included into the analysis had the following ages (in month) and HSRC presentations (or clinical presentations for Non-HSCR): Fiber-low: 3/S-HSCR; 5/L-HSCR; 3/S-HSCR. Fiber-high: 3/S-HSCR; 4/S-HSCR; 3/S-HSCR. CD G (Non-HSCR): 5/anal atresia; 6/anal atresia; 7/anal atresia.

### Stimulation of SW480 Cells

The human epithelial colorectal adenocarcinoma cell line SW480 (ATCC, LGC Standards, France) was maintained in Dulbecco’s Modified Eagle’s Medium (DMEM, ThermoFisher Scientific, Waltham, MA, USA) supplemented with 10% FCS and 1% Penicillin-Streptomycin (Merck, Darmstadt, Germany) under humidifying conditions (5x10^4^-x10^5^ cells/ml at 37°C and 5% CO_2_). For cytokine induction cells were seeded into 96-well flat-bottom plates (2.5 x 10^5^/ml, Corning, New York, NY, USA). Next day, the medium was exchanged, and cells were stimulated with 100ng/ml lipopolysaccharide (LPS, *E.coli*, Merck, Darmstadt, Germany) in the presence or absence of agonists and antagonists/inhibitors. Antagonists and inhibitors were added 20min prior to AChR agonists and thus 20min prior to LPS stimulation. The following agonists/antagonists were used: acetylcholine chloride (10µM, AChR agonist, Merck, Darmstadt, Germany), nicotine (10µM, nAChR agonist, Merck, Darmstadt, Germany), muscarine chloride hydrate (10µM, mAChR agonist, Merck, Darmstadt, Germany), GTS-21 (100µM, a7nAChR agonist, Merck, Darmstadt, Germany), mecamylamine HCL (100µM, nAChR antagonist, Merck, Darmstadt, Germany), alpha-Bungarotoxin (100µM, a7nAChR antagonist, Alomone labs, Jerusalem, Israel), tyrphostin AG490 (1µM, blocking Janus kinase (Jak) 2, Merck, Darmstadt, Germany), and wortmannin (1µM, blocking phosphoinositide 3-kinase (PI3K), Merck, Darmstadt, Germany). After 20h, cell-free supernatants were collected for IL-8 analysis and cell viability was analyzed using CellTiter 96 Aqueous One Solution Cell Proliferation Assay (MTS; Promega, Dubendorf, Switzerland). For phospho (p) NFĸB -p65 and RNA extraction cells were stimulated for 4h prior to cell lysis. Supernatants and cell lysates were stored at -80.

### Human IL-8 and Phospho NFĸB-p65 ELISA

IL-8 was determined in cell culture supernatants and IEC lysates using a human IL-8 ELISA Ready-SET-Go Kit (eBioscience, San Diego, CA, USA) according to the instruction manual. Patient-derived IEC were lysed in Cell Extraction Buffer (5 x 10^5^ cells/100µl, ThermoFisher Scientific, Waltham, MA, USA) containing protease inhibitor cocktail (Merck, Darmstadt, Germany). Patients included into the analysis had the following ages (in month) and HSRC presentations: Fiber-low: 3/S-HSCR; 5/L-HSCR; 3/S-HSCR; 3/S-HSCR; 2/S-HSCR. Fiber-high: 3/S-HSCR; 4/S-HSCR; 3/S-HSCR; 3/S-HSCR; 4/L-HSCR. CD AG: 5/TCA; 11/L-HSCR; 5/TCA; 5/L-HSCR. CD G: 3/S-HSCR; 5/S-HSCR; 2/S-HSCR.

Phospho (p)-p65 was determined in SW480 cells using a human NFĸB p65 Human InstantOne™ ELISA Kit (ThermoFisher Scientific, Waltham, MA, USA) according to the instruction manual. ELISA plates were measured using a Synergy H4 Hybrid Reader and GEN5 2.00 software (BioTeK).

### Statistical Analysis

Data were analyzed using Prism GraphPad 6.0 software. Data are reported as the mean ±standard error of the mean (SEM). Statistical significance was determined using the Kruskal-Wallis test, one-way ANOVA multiple comparison analysis as well as paired t test and considered statistically significant with * p≤ 0.05; ** p≤ 0.01; *** p≤ 0.001 and **** p≤ 0.0001. Unless otherwise noted, figures showed pooled patient data from several independent experiments or a representative of independent repeated experiments tested in triplicates.

## Results

### Colonic Mucosal Innervation and Characteristics of HSCR Patients

**Figure 1:**
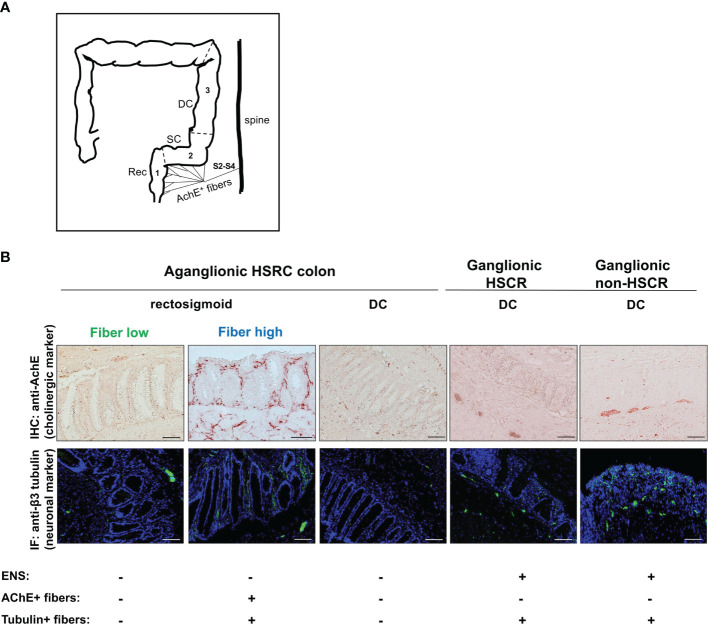
Neuronal innervation of the lower colon track in HSCR and non-HSCR patients. Schematic representation of extrinsic AChE^+^ nerve fiber innervation **(A)**. Anatomically, extrinsic innervation derived from sacral roots S2–4 is limited to the rectosigmoid and does not reach the descending colon. Rec (1), rectum; SC (2), sigmoid colon; DC (3), descending colon; AChE, acetylcholinesterase; S2–4, sacral roots S2–4. Cryosections (5µm) of aganglionic and ganglionic colonic tissue from HSCR and non-HSCR patients showing anti-AChE immunohistochemistry and anti-ß3 tubulin immunofluorescence **(B)**. Five different colonic tissue types were distinguished, and each showed different expression of ENS (aganglionic versus ganglionic) and mucosal nerve fiber innervation. Scale bar: 50µm. IHC, immunohistochemistry; IF, immunofluorescence; ENS, enteric nervous system.

We enrolled 43 pediatric HSCR patients, undergoing therapeutic pull-through surgery, as well as 6 control patients (non-HSCR), undergoing surgery for miscellaneous reasons, from the prospective, multicenter NIG (Neuroimmune Interactions in the Gut) study. In rectosigmoid tissue of non-HSCR patients AChE activity is limited to the myenteric plexus located between the longitudinal and transverse muscle layers (5). The aganglionic colons of HSCR patients lack the myenteric and submucosal plexus but show the characteristic prominent AChE activity in the rectosigmoid. In the muscularis and submucosa, thick AChE^+^ fiber bundles can be detected while the mucosa is innervated by thin fiber ramification. We recently discovered that HSCR patients differ in the density of mucosal cholinergic innervation in the rectosigmoid and established a semi-quantitative scoring system (4). This allowed us to allocate HSCR patient tissue into fiber-low and fiber-high tissue phenotypes according to the presence of thin fibers in the mucosa of the rectosigmoid (4). Of note, increased cholinergic activity is limited to the aganglionic rectosigmoid and decreases in caudocranial direction. As a consequence, no extrinsic mucosal AChE^+^ nerve fibers can be detected in the proximal aganglionic descending colon (DC) (**Figure 1A**) (5). Depending on the length of aganglionosis, the proximal DC segment of HSCR patients can be ganglionic or aganglionic. Consequently, for further analysis, aganglionic and ganglionic segments with variable mucosal innervation were defined as illustrated by anti-AChE (cholinergic marker) and anti-β3 tubulin (pan neuronal marker) immunohistochemistry (**Figure 1B**). Aganglionic rectosigmoid fiber-low tissues were those lacking an intrinsic ENS and showing only sparse mucosal extrinsic AChE^+^ innervation. Aganglionic rectosigmoid fiber-high tissues were those lacking an intrinsic ENS but showing dense mucosal extrinsic AChE^+^ innervation. Aganglionic DC tissues lacked an intrinsic ENS as well as extrinsic mucosal innervation due to anatomical restriction of AChE^+^ nerve fibers to the rectosigmoid. Ganglionic DC sections from HSCR and non-HSCR patients showed the presence of intrinsic AChE^+^ enteric ganglia located in the muscle region (asterisk) and tubulin^+^ (arrow) but AChE^-^ nerve fibers in the mucosa and submucosa (**Figure 1B**) (4).

**Table 1** summarizes the demographic and clinical parameters of the patient cohort. The median age of HSCR patients was 5.7 months with a range of 1–127.5, while the median age of non-HSCR patients was 6.35 months with a range of 0.4–11.9. Male patients were more common in HSCR (35:8), while the male to female ratio was 5 to 1 in non-HSCR. Ten clinics participated in the study and 5 different surgical approaches were used for the surgical treatment of HSCR patients. Most frequently the transanal Soave technique was applied (n=34), while other surgical techniques were only used occasionally. HSCR patients presented with ultrashort or short-segmented (n=28), long-segmented (n=8) and total (n=7) colonic aganglionosis. Of our 43 HSCR patients, 13 (30%) showed a fiber-high tissue phenotype in the aganglionic rectosigmoid segments while the other 30 patients were categorized as fiber-low (70%). Descending colon tissue was available from 22 patients: 10 tissue samples (45%) were aganglionic (8 from fiber-high and 2 from fiber-low patients) and 12 (55%) were ganglionic (equally distributed among fiber-high and fiber-low patient tissue).

**Table 1:** Patient characteristics.

Patient cohort characteristics	HSCR	non-HSCR
Total (n)	43	6
**Age (month)**		
Mean	14	6.2
Median	5.7	6.35
Range	1–127.5	0.4–11.9
**Sex**		
Male	35 (81%)	5 (83%)
Female	8 (19%)	1 (17%)
**Clinic**		
Basel (CH)	4 (9%)	4 (67%)
Bern (CH)	4 (9%)	0
Düsseldorf (DE)	2 (5%)	0
Freiburg (DE)	3 (7%)	0
Geneva (CH)	1 (2%)	0
Heidelberg (DE)	3 (7%)	1 (17%)
Karlsruhe (DE)	4 (9%)	0
Lausanne (CH)	5 (12%)	0
St. Gallen (CH)	6 (14%)	0
Zürich (CH)	11 (26%)	1 (17%)
**Surgical technique**		
Swenson transanal	3 (7%)	
Soave transanal	34 (79%)	
Swenson transabdominal	1 (2%)	
Soave transabdominal	4 (9%)	
Duhamel transanal	1 (2%)	
**Length of aganglionosis**		
Ultrashort & short	28 (65%)	
Long	8 (19%)	
TCA	7 (16%)	
**Fiber phenotype**		
Fiber-high	13 (30%)	
Fiber-low	30 (70%)	
**Descending colon control tissue**		
Total aganglionic DC	10 (23%)	
from fiber-high patient	8 (19%)	
from fiber-low patient	2 (5%)	
Total ganglionic DC	12 (28%)	
from fiber-high patient	6 (14%)	
from fiber-low patient	6 (14%)	

Characteristics of prospective HSCR patient cohort. Values are absolute numbers (percentage %) for categorical variables and mean/median/range for continuous variables. HSCR, Hirschsprung disease; CH, Switzerland; DE, Germany; TCA, total colonic aganglionosis; DC, descending colon.

### Lack of Mucosal Cholinergic Fibers Is Associated With Elevated IL-8 in IEC

**Figure 2:**
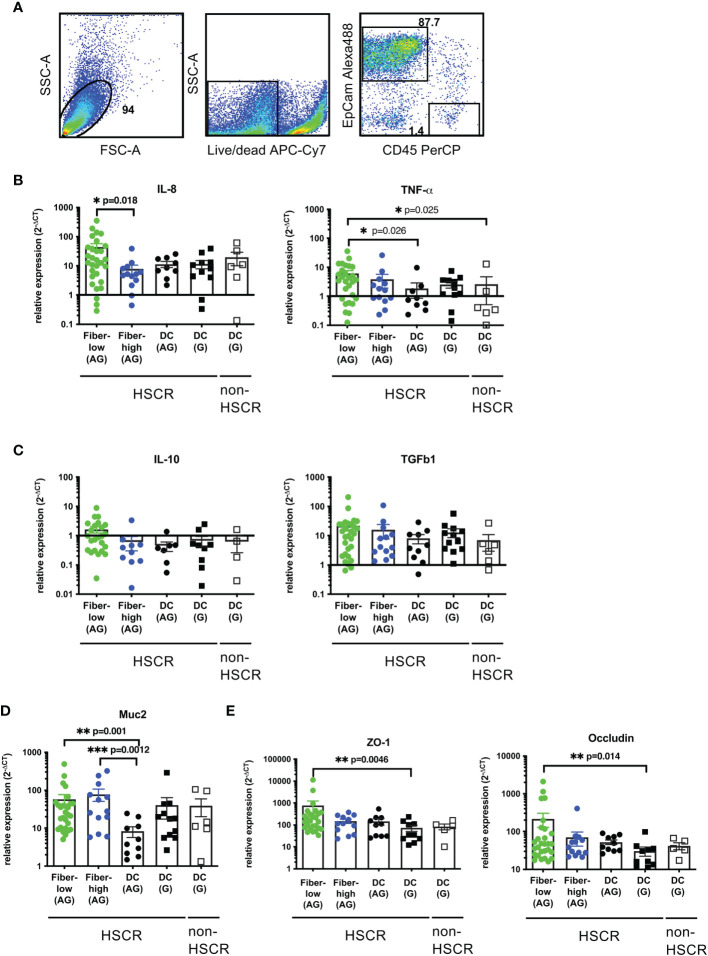
Expression of cytokines, mucin, and tight junctions in patient-derived IEC. FACS analysis of isolated IEC. Cells were isolated by digestion and purified using a 20%/40% Percoll gradient. Purity was determined by flow cytometric analysis using antibodies against human CD45-PerCP and EpCam-Alexa488. Unstained controls were used to determine the negative population **(A)**. Purified epithelial cells were immediately lysed, total RNA isolated and cDNA synthesized. Relative gene expression was calculated using the 2-ΔCT method, with β2-microglobulin as the housekeeping gene **(B–E)**. Scatter plots with bar show mean ± SEM. Significance was determined using the Kruskal-Wallis test (*p ≤ 0.05, **p ≤ 0.01, ***p ≤ 0.001). Fiber-low (n=30); Fiber-high (n=13); DC AG (n=10); DC G, HSCR (n=12); DC G, non-HSCR (n=6). Not detectable samples: Fiber-low: TNF-α (n=2); IL-10 (n=6); TGF-*β* (n=1); Muc2 (n=2); ZO-1 (n=4); occludin (n=2). Fiber-high: IL-10 (n=4); ZO-1 (n=1). DC AG: IL-8 (n=1); TNF-α (n=1); IL-10 (n=3). DC G, HSCR: IL-8 (n=1); TNF-α (n=1); IL-10 (n=3); ZO-1 (n=2); occludin (n=2). DC G, non-HSCR: IL-10 (n=2); ZO-1 (n=1); occludin (n=1). DC, descending colon; AG, aganglionic; G, ganglionic.

To investigate a possible correlation between mucosal AChE^+^ fiber density and IEC cytokine production we performed Quantitative Real-Time Polymerase Chain Reaction (qRT-PCR) analysis of isolated IEC from surgically removed colonic tissue of HSCR and non-HSCR patients. IEC purity (EpCam^+^CD45^-^cells) was confirmed by FACS analysis (**Figure 2A**).

IEC were tested for the expression of pro-inflammatory (IL-8 and TNFα) and anti-inflammatory cytokines (IL-10 and TGFβ1). In aganglionic rectosigmoid tissues, IEC isolated from fiber-low segments expressed significantly higher IL-8 levels compared to the fiber-high group (**Figure 2B**). No differences were detected between aganglionic and ganglionic (HSCR and non-HSCR) DC segments. TNFα expression was significantly increased in fiber-low segments compared to DC control segments (**Figure 2B**). For IL-10 and TGFβ1 expression no significant difference could be observed between the groups (**Figure 2C**).

Next, we investigated a possible impact of neuronal innervation on mucus secretion and tight junction proteins. The human mucus barrier consists mainly of MUC2 and is produced by specialized epithelial cells, the goblet cells. We observed an increased expression of MUC2 in IEC isolated from fiber-low and fiber-high colonic segments compared to aganglionic DC (**Figure 2D**). The tight junction proteins zona occludens-1 (ZO-1) and occludin play critical roles in maintaining the epithelial barrier to prevent invasion of luminal microbes. We observed increased expression of ZO-1 and occludin in IEC isolated from fiber-high compared to ganglionic DC and aganglionic DC, respectively (**Figure 2E**) (4).

**Figure 3:**
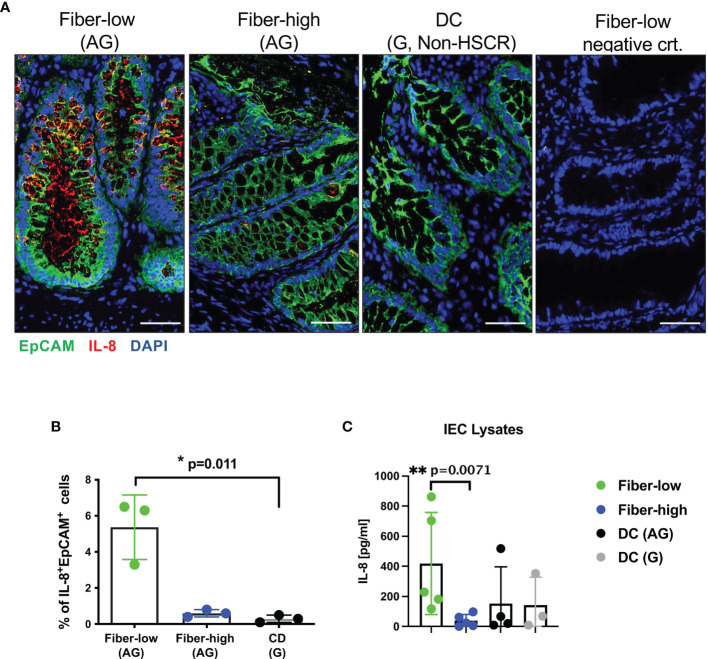
IL-8 protein expression in patient-derived IEC. Immunofluorescence (5µm cryosections) of rectosigmoid using human antibodies against EpCAM (Alexa488, green) and IL-8 (Alexa555, red). DAPI (blue) shows cell nuclei. Secondary antibody goat anti-mouse IgG1 Alexa 555 was used as the negative control. Scale bars 50µm **(A)**. Quantification of IL-8 using Cellprofiler-3.1.9 software. IL-8 expression in EpCAM^+^ IEC (n=3) was quantified in 4 to 8 epithelial regions (on average 1500 IEC) per patient from fiber-low and fiber-high rectosigmoid tissue. Frequencies of IL-8^+^ EpCam^+^ cells were shown as the percentage of total EpCam^+^ epithelial cells. **(B)**. Patient-derived IEC were lysed, and IL-8 was determined using human IL-8 ELISA Ready-SET-Go Kit. **(C)** Significance was determined using the Kruskal-Wallis test (*p ≤ 0.05, **p ≤ 0.01). Scatter plots show means ± SEM. AG, aganglionic; G, ganglionic; DC, descending colon.

To investigate IL-8 protein levels, we performed immunofluorescence analysis on cryosections from aganglionic rectosigmoid fiber-low, fiber-high and DC segments. Representative images of epithelial crypt regions show prominent IL-8^+^EpCAM^+^ IEC in rectosigmoid tissue from fiber-low but not from fiber-high and DC control tissue (**Figure 3A**). We quantified IL-8^+^EpCAM^+^ IEC in 4–8 epithelial regions (containing on average 1500 IEC) of 3 fiber-low and 3 fiber-high patient tissue samples using the Cellprofiler-3.1.9 software. We detected increased frequencies of IL-8-positive IEC in fiber-low compared to fiber-high and DC tissue (**Figure 3B**). In addition, we detected IL-8 protein levels in IEC cell lysates and found significantly higher IL-8 concentrations in IEC derived from fiber-low compared to fiber-high or aganglionic DC segments (**Figure 3C**). In conclusion, the presence of mucosal AChE^+^ nerve fibers correlated with decreased IL-8 expression in IEC, while mucus production and tight junctions were not affected.

### IEC From Distal Aganglionic Colon Express Several AChRs

**Figure 4:**
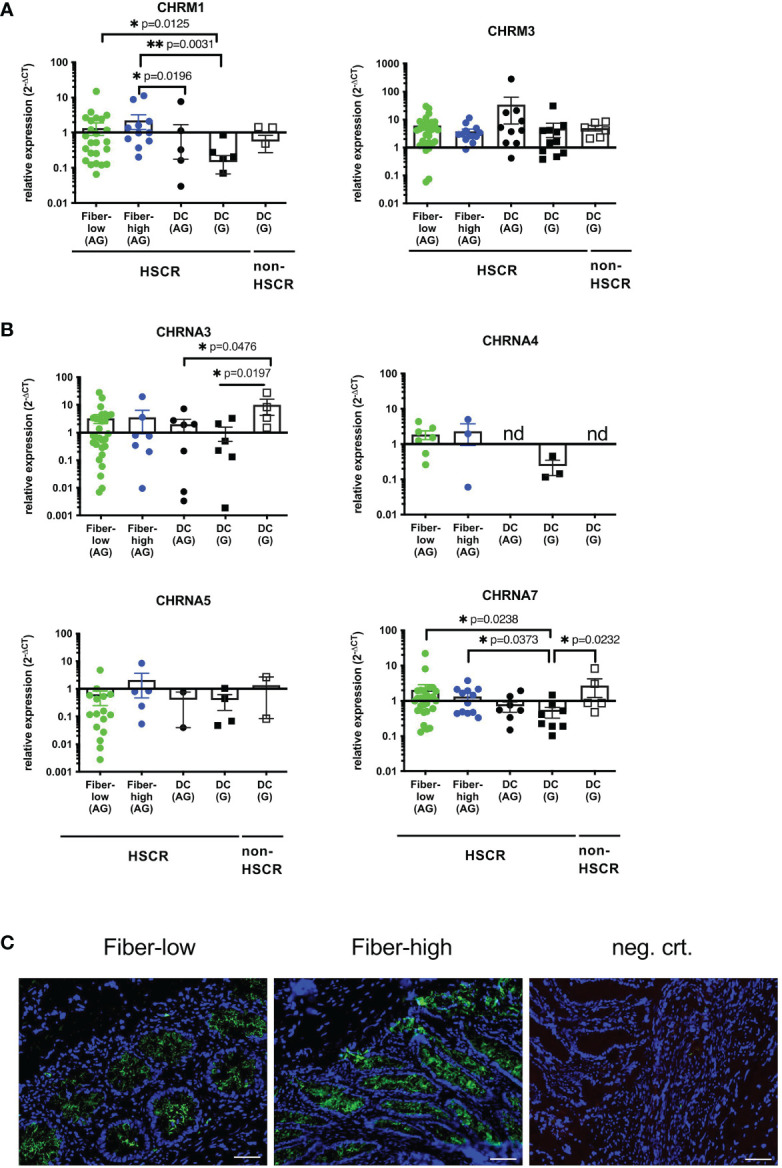
Expression of IEC-specific cholinergic receptors. Purified epithelial cells were immediately lysed, total RNA isolated and cDNA synthesized. Relative gene expression was calculated using the 2-ΔCT method, with β2-microglobulin as the housekeeping gene **(A, B)**. Scatter plots with bar show mean ± SEM. Significance was determined using the Kruskal-Wallis test (*p ≤ 0.05, **p ≤ 0.01). Fiber-low (n=30); Fiber-high (n=13); DC AG (n=10); DC G, HSCR (n=12); DC G, non-HSCR (n=6). Not detectable samples: Fiber-low: CHRM1 (n=1); CHRNA3 (n=2); CHRNA4 (n=23); CHRNA5 (n=14); CHRNA7 (n=2). Fiber-high: CHRNA3 (n=6); CHRNA4 (n=10); CHRNA5 (n=8); CHRNA7 (n=1). DC AG: CHRNA3 (n=3); CHRNA4 (n=10); CHRNA5 (n=8); CHRNA7 (n=2). DC G, HSCR: CHRM1 (n=1); CHRM3 (n=1); CHRNA3 (n=6); CHRNA4 (n=9); CHRNA5 (n=8); CHRNA7 (n=4). DC G, non-HSCR: CHRNA3 (n=2); CHRNA4 (n=6); CHRNA5 (n=4); CHRNA7 (n=1). nd: not detectable. CHRNA7 Immunofluorescence (5µm cryosections) from fiber-low and fiber-high rectosigmoid using purified mouse IgG1 anti-alpha7nAchR antibody (green) **(C)**. DAPI (blue) shows cell nuclei. Secondary antibody goat anti-mouse IgG1 Alexa 555 was used as the negative control. Scale bar 50µm.

We aimed to identify IEC-specific AChRs possibly involved in neuronal modulation. According to the literature, intestinal epithelial cells express preferentially muscarinic M1 (CHRM1) and M3 (CHRM3) receptors as well as nicotinic α3 (CHRNA3), α4 (CHRNA4), α5 (CHRNA5) and α7 (CHRNA7) subunits (38). We performed qRT-PCR on isolated patient-derived IEC and checked for the expression of the receptors. Among the muscarinic receptors CHRM1 was less prominently expressed than CHRM3 (**Figure 4A**). IEC isolated from fiber-low and fiber-high rectosigmoid showed higher expression of CHRM1 compared to ganglionic HSCR DC tissue. Further, CHRM1 expression was significantly higher in IEC isolated from fiber-high tissue compared to aganglionic DC tissue. No difference in CHRM3 expression was observed between the different groups (**Figure 4A**). Among nicotinic subunits, the CHRNA4 and CHRNA5 were expressed in lower levels than CHRNA3 and CHRNA7 and not detectable in most of the samples (see figure legend) (**Figure 4B**). We could not detect significant differences for CHRNA4 and CHRNA5 expression between the diverse sample groups. CHRNA3 expression was significantly elevated in ganglionic DC samples from non-HSCR patients compared to aganglionic and ganglionic DC samples from HSCR patients. Interestingly, the CHRNA7 expression was significantly higher in samples from rectosigmoid fiber-low and fiber-high samples compared to ganglionic DC samples. In conclusion we found elevated expression levels of the muscarinic M1 and nicotinic alpa 7 AChRs in IEC isolated from aganglionic fiber-low and fiber-high rectosigmoid compared to ganglionic control tissue. Using anti-a7AChR immunofluorescence we further demonstrated comparable protein expression in epithelial crypts of fiber-low and fiber-high colonic HSCR tissue (**Figure 4C**).

### Cholinergic Stimulation Attenuates LPS-Induced IL-8 Response in SW480 Cells *via* a7nAChR

**Figure 5:**
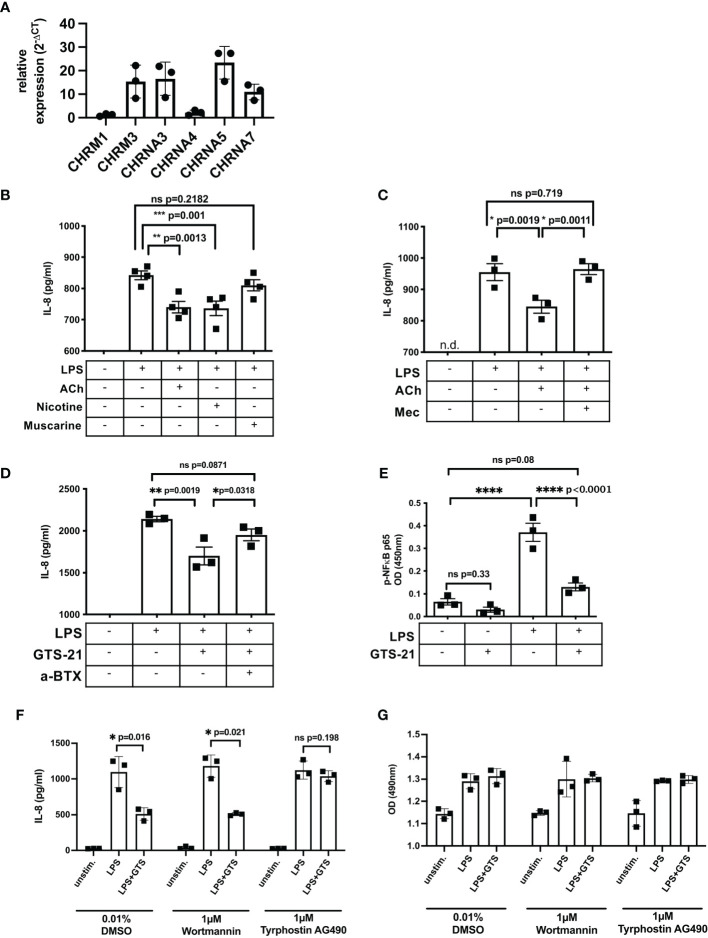
Cholinergic stimulation attenuates LPS-induced IL-8 response in SW480 cells *via* a7nAChR. Expression of ACHRs in SW480 cells **(A)**. SW480 cells (5 x 10^4^/200µl) were stimulated with LPS (100ng/ml) in the presence of different agonist and antagonists. IL-8 was measured in cell-free supernatants 20h after LPS stimulation. Cells were stimulated with LPS 20min prior to acetylcholine (ACh, 10µM), nicotine (10µM), and muscarine (10µM) stimulation **(B)**. Cells were pretreated (20min) with the non-selective nAChR antagonist Mecamylamine (Mec, 100µM) followed by treatment with ACh (10µM), 20min prior to LPS stimulation **(C)**. Cells were pretreated (20min) with the selective a7nAChR antagonist alpha-Bungarotoxin (a-BTX, 100µM) followed by treatment with GTS-21 (100µM), 20min prior to LPS stimulation **(D)**. Cells were pretreated (20min) with GTS-21 (100µM) followed by stimulation with LPS. Phospho (p)-NFĸB-p65 was measured 4h after LPS stimulation **(E)**. Cells were pretreated (20min) with DMSO (0.01%, control) or tyrphostin AG490 (1µM) or wortmannin (1µM) followed by stimulation with LPS (100ng/ml), 20min prior to treatment with a7nAChR agonist GTS-21 (20min) **(F)**. Cell viability was assessed by colorimetric MTS assay **(G)**. Each point in the scatter plots with bar represent triplicates or quadruplicates ± SEM. Data are representative of three independent experiments. Significance was determined using one-way ANOVA multiple comparison analysis **(A–D)** and paired t test **(E, F)** with *p ≤ 0.05; **p ≤ 0.01; ***p ≤ 0.005. ns, not significant. n.d., not detectable.

To identify the cholinergic receptor involved in suppression of epithelial IL-8 response, we performed functional studies using the human colorectal adenocarcinoma cell line SW480. The expression pattern of ACHRs was comparable to patient-derived IEC except the expression of CHRNA5 which was only weakly expressed in IEC (**Figure 5A**). To induce an IL-8 response, SW480 cells were stimulated with Lipopolysaccharide (LPS), a component of the outer membrane of gram-negative bacteria, and supernatants were tested for IL-8 concentrations using ELISA. First, cells were stimulated with LPS in the presence of acetylcholine (stimulating both, mAChR and nAChRs), nicotine (activating nACHRs) or muscarine (activating mAChRs) (**Figure 5B**). Compared to muscarine, the presence of acetylcholine or nicotine significantly decreased LPS-induced IL-8 response (**Figure 5B**). Further, the suppressive effect of acetylcholine was reversed by mecamylamine, a non-selective nAChR antagonist, suggesting nicotinic receptor involvement (**Figure 5C**). The a7nAChR has been described to suppress inflammatory cytokine production in several cell types (39). Since we found a significant elevation of a7nAChR in IEC isolated from the distal aganglionic colon we tested its involvement in regulation of LPS-induced IL-8 response. We stimulated SW480 cells with LPS in the presence of GTS-21, a selective a7nAChR agonist (**Figure 5C**). Addition of GTS-21 significantly decreased LPS-induced IL-8 response, which could be reversed by adding α-Bungarotoxin, a selective a7nAChR antagonist (**Figure 5D**). LPS-induced IL-8 production is induced *via* the activation and phosphorylation of the transcription factor subunit nuclear factor ‘kappa light chain enhancer’ of activated B cells (NF-ĸB) p65 (40, 41). We stimulated SW480 cells with LPS in the presence and absence of GTS-21 and measured phosho (p)-p65 in cell lysates (**Figure 5E**). LPS stimulation induced phosphorylation of p65 which significantly decreased in the presence of GTS-21. Dependent on the cell type, the JAK2/signal transducer and activator of transcription 3 (Stat3) as well as the PI3K/protein kinase B (Akt) signaling pathway have been reported to mediate the cholinergic anti-inflammatory mechanism (42–48). To identify involved signaling components leading to LPS-induced IL-8 suppression after a7nAChR stimulation we used selective inhibitors specific for Jak2/3 (tyrphostin AG490) and PI3K (wortmannin). GTS-21-mediated suppression of IL-8 was unaffected in the presence of wortmannin but was abrogated in the presence of tyrphostin AG490 (**Figure 5F**) while cell viability was not affected by the inhibitors (**Figure 5G**). These results suggest that activation of the a7AChR attenuated LPS-induced IL-8 in SW480 cells involving activation of the JAK2/3 signaling pathway.

## Discussion

Here, we classified the aganglionic rectosigmoid of HSCR patients dependent on a prominent (fiber-high) or sparse (fiber-low) mucosal cholinergic innervation and showed that the presence of cholinergic innervation is associated with decreased pro-inflammatory IL-8 levels in patient-derived IEC. Muscarinic 1 as well as nicotinic alpha-7 AChRs were upregulated in IEC isolated from aganglionic rectosigmoid compared to IEC isolated from ganglionic control tissue. Using the human IEC cell line SW480, we demonstrated that activation of a7nAChR suppressed LPS-induced IL-8 response involving the JAK2/3 signaling molecule.

IL-8 is a classical marker of colonic inflammation secreted in response to inflammatory cytokines or bacterial stimuli (33, 49). It is mainly produced by epithelial/endothelial cells, monocytes, and fibroblasts to recruit neutrophils to inflammatory sites. Daig and colleagues demonstrated increased IL-8 expression in the colonic mucosa of inflammatory bowel disease (IBD) patients (34). However, they identified IL-8-positive cells in the lamina propria but not in epithelial cells. We have clearly demonstrated an IEC-specific expression of IL-8 on both, at mRNA and protein level. We used an isolation protocol based on density gradient typically reaching a purity of EpCAM^+^ cells of 90% with less than 2% contamination with CD45^+^ cells. Using qRT-PCR and ELISA analysis we showed elevated expression as well as protein levels in IEC isolated from fiber-low compared to fiber-high aganglionic rectosigmoid. IL-8 immunofluorescence verified the expression in EpCAM^+^ IEC. We found elevated TNF-α levels in IEC derived from fiber-low compared to aganglionic and ganglionic DC. Comparable expression levels among all groups were detected for IL-10 and TGF-β. Borovikova’s study on the effect of CAIP in LPS-stimulated human macrophages showed that cholinergic signaling preferentially acts on the diminishment of pro-inflammatory cytokines without affecting the anti-inflammatory response (24, 25, 50). We have detected elevated expression of Muc2 as well a tight junction proteins ZO-1 and occludin in fiber-low tissue excluding an alteration in mucus production and epithelial barrier function. In the literature, alteration in mucin composition and turnover was reported as leading to altered epithelial barrier function and consequently to a higher risk of developing HSCR-associated enterocolitis (HAEC) (16, 51–53). In fact, in HSCR patients developing HAEC, mucus production and turnover is markedly reduced compared to HSCR patients without HAEC (54, 55). A recent study using a piglet model of chemical-induced aganglionosis showed increased epithelial permeability, with decreased ZO-1 protein expression, in hypoganglionic colonic segments (56). However, it is not clear if the reported alterations in mucus and tight junction expression is due to a direct or indirect effect of underlying aganglionosis. Further, a decrease in tight junction proteins might be only detectable prior to or at the time of enterocolitis onset.

As possible receptors mediating cholinergic signals, we identified CHRNA7 and CHRM1 in patient-derived IEC. Expression levels were elevated in IEC isolated from aganglionic rectosigmoid of both fiber-low and fiber-high phenotypes, compared to IEC isolated from ganglionic descending colon specimens. Cholinergic signals are critical in controlling epithelial ion transport (23), but also contribute to immune homeostasis and barrier function (57). Gahring et al. showed that smoking (nicotine) exerts an anti-inflammatory modulation of lung epithelium in mice—an effect that seems to be mediated *via* the a7nAChR (58). Other authors investigated the effect of cholinergic signaling on colitis: Lakhan et al. postulated that the anti-inflammatory effect of smoking on ulcerative colitis was due to nicotine signaling involving the a7nAChR (59). Muscarinic receptor activation protects colonic epithelial cell lines from cytokine-induced barrier dysfunction (60, 61) and increases epithelial cell layer tightness (62).

To identify possible underlying mechanisms of cholinergic regulation of epithelial IL-8 secretions we performed functional studies using the human colonic epithelial cell line SW480. Using selective agonists and antagonists for muscarinic, nicotinic and a7 nicotinic AChRs, we identified the a7nAChR as mediating the cholinergic anti-inflammatory effect on LPS-induced IL-8 secretion. The a7nAChR has been described as the main receptor mediating the cholinergic anti-inflammatory effect by decreasing NF-ĸB activity followed by lower cytokine response (39). LPS binds to Toll-like receptor 4 (TLR4) to activate the transcription factor NF-ĸB leading to secretion of inflammatory IL-8. Several signaling pathways contribute to NF-ĸB inhibition after a7nAChR activation. In monocytes/macrophages a7nAChR signaling involves the activation of the JAK2-STAT3 signaling pathway (43, 44). Dependent on the cell type, several signaling cascades in response to a7nAChR have been suggested, mainly including the activation of negative regulators of TLR signaling. Myeloid differentiation factor 88 (MyD88) is an adaptor protein of TLR receptors essential for the initiation of downstream signaling. In HBE16 airway epithelial cells, nicotine decreased the expression of MyD88 and subsequent NF-ĸB activation (63). MyD88 is associated with Interleukin-1 receptor-associated kinases (IRAK) 1 and 4, which after activation dissociate from MyD88 to activate TNF receptor-associated factor 6 (TRAF6). In human macrophages, Maldifassi et al. uncovered that nicotine triggers IRAK-M activation, preventing the dissociation of active IRAKs from MyD88 (39, 64). Another negative regulator of the TLR4 signaling pathway is PI3K (65). In a murine model of LPS-induced neuroinflammation, treatment with Donepezil—an inhibitor of ACh degrading enzyme acetylcholinesterase—improved sepsis survival. The effect was mediated *via* PI3K-Akt activation leading to decreased TLR4 expression and lower inflammatory cytokine production (46). In renal inflammation, proximal tubular a7nAChR activation protected against acute kidney injury and tubular cell death through PI3K-Akt and PKC signaling (47). Xue et al. demonstrated in a model of neuroinflammation that administration of a7nAChR agonist induced activation of PI3K-Akt to reduce inflammatory cytokine response (48). Using human intestinal epithelial cells, we showed that GTS-21-mediated suppression of LPS-induced IL-8 in SW480 cells was sensitive to tyrphostin AG490 treatment, assuming the involvement of JAK2/3 in a7nAChR downstream signaling. However, further a7nAChR signaling pathway molecules remain to be identified. Interestingly, inflammatory mediators induce the upregulation of a7nAChR to counter-regulate pro-inflammatory cytokines and protect the host from excessive inflammation and tissue pathology (66, 67).

HAEC is the most frequent life-threatening complication in HSCR, both before and after therapeutic pull-through surgery. However, currently no targeted therapies for HAEC are available. A hallmark of HAEC histopathology is the infiltration of neutrophils and the subsequent initiation of a TH17-mediated immune response (1, 4). IL-8 is a chemoattractant for neutrophils and consequently is essential in the induction of early leukocyte recruitment and inflammatory processes. Increased levels of IL-8 have been described in the mucosa of IBD patients (34, 68, 69) and IL-8 gene polymorphism is associated with IBD predisposition (36, 70). We found elevated IL-8 levels in IEC isolated from the rectosigmoid of HSCR patients showing a fiber-low compared to a fiber-high phenotype. Recently, we demonstrated in the same HSCR patient cohort that enterocolitis was more prevalent in patients with a sparse mucosal nerve fiber innervation density (4). After one year, 9 out of 42 HSCR patients developed HAEC, of which 7 patients had a fiber-low (78%) and 2 a fiber-high (22%) phenotype. The results were validated in a large retrospective HSCR cohort including 104 patients (Moesch et al., under review). Therefore, IL-8 might serve as a novel target for early HAEC therapy (71). Currently, two IL-8 targeting antibodies, ABX-CXCL8 and HuMax-CXCL8, are being used in preclinical studies including patients with severe bronchitis and chronic obstructive pulmonary disease (COPD) (NCT00035828) as well as patients with advanced malignant solid tumors (NCT02536469; NCT03689699) (72). However, in this study we analyzed IL-8 at the time of pull-through surgery when none of the patients had yet experienced an enterocolitis. Due to limited cases of HAEC, further investigations and validations are needed to confirm an association of IL-8 levels with the onset of enterocolitis.

We suggest that the presence of neuronal cholinergic innervation in the colon might help to maintain a tolerogenic anti-inflammatory immune cell status to prevent excessive immune responses and tissue pathology. We recently showed that the fiber-high phenotype correlated with reduced inflammatory T-helper-17 cytokines and cell frequencies (4). In fiber-high tissue, nerve fibers colocalized with anti-inflammatory macrophages expressing significantly less interleukin-23 (a TH17-inducing cytokine) than macrophages from fiber-low tissue. It has been reported that colonic inflammatory TH17 cells migrate to neighboring mesenteric lymph nodes, triggering subsequent enterocolitis development (73). Since the mesentery is generally not removed during surgery and reconnects to the neo rectosigmoid, primed immune cells might repopulate the tissue possibly explaining why patients can develop enterocolitis even after removal of the affected aganglionic colon segments.

In summary, we have shown for the first time that the presence of mucosal cholinergic innervation is associated with decreased secretion of IEC-derived pro-inflammatory IL-8 in the rectosigmoid of HSCR patients likely dependent on activation of a7nAChR. Since patients lacking a dense mucosal cholinergic innervation are at high risk of developing enterocolitis, these results suggest a possible involvement of IL-8 in the early onset of HAEC, presenting IL-8 as a novel target for immunotherapy.

## NIG Study Group

Isabella Bielicki^1^, Sandra Weih^1^, Noëmi Zweifel^2^, Kai-Uwe Kleitsch^3^, Carole Gengler^9^, Kiarasch Mortazawi^5^, Milan Milosevic^6^, Vera Otten^8^, Lennart Homrighausen^8^

## Data Availability Statement

The raw data supporting the conclusions of this article will be made available by the authors, without undue reservation.

## Ethics Statement

The studies involving human participants were reviewed and approved by the North-West Switzerland Ethics Commission (EKNZ 2015-049) and the Medical Faculty of Heidelberg (S-388/2015). Written informed consent to participate in this study was provided by the participants’ legal guardian/next of kin.

## Author Contributions

Concept and design: SK. Acquisition of data: IM, UK, VG, ST, US, TK, ES, PS, DC, PR, BR, SH-C, SK, and the members of the NIG study group. Analysis and interpretation of data: IM, UK, VG, and SK. Drafting of the manuscript: IM and SK. Revision of the manuscript: all authors. All authors contributed to the article and approved the submitted version.

## Funding

This work was supported by the Stay on Track funding (awarded to SK) and the Research Fund Junior Researcher (awarded to SK; DMS2343) from the University of Basel, Switzerland.

## Conflict of Interest

The authors declare that the research was conducted in the absence of any commercial or financial relationships that could be construed as a potential conflict of interest.

## Publisher’s Note

All claims expressed in this article are solely those of the authors and do not necessarily represent those of their affiliated organizations, or those of the publisher, the editors and the reviewers. Any product that may be evaluated in this article, or claim that may be made by its manufacturer, is not guaranteed or endorsed by the publisher.
